# Lack of Spatial Immunogenetic Structure among Wolverine (*Gulo gulo*) Populations Suggestive of Broad Scale Balancing Selection

**DOI:** 10.1371/journal.pone.0140170

**Published:** 2015-10-08

**Authors:** Yessica Rico, James Morris-Pocock, Joanna Zigouris, Joseph J. Nocera, Christopher J. Kyle

**Affiliations:** 1 Forensic Science Department, Trent University, Peterborough, ON, Canada; 2 Natural Resources DNA Profiling and Forensics Centre, Trent University, Peterborough, ON, Canada; 3 Environmental and Life Sciences Graduate Program, Trent University, Peterborough, ON, Canada; 4 Applied Research Development Branch, Wildlife Research Development Section, Ministry of Natural Resources, Peterborough, ON, Canada; University of British Columbia Okanagan, CANADA

## Abstract

Elucidating the adaptive genetic potential of wildlife populations to environmental selective pressures is fundamental for species conservation. Genes of the major histocompatibility complex (MHC) are highly polymorphic, and play a key role in the adaptive immune response against pathogens. MHC polymorphism has been linked to balancing selection or heterogeneous selection promoting local adaptation. However, spatial patterns of MHC polymorphism are also influenced by gene flow and drift. Wolverines are highly vagile, inhabiting varied ecoregions that include boreal forest, taiga, tundra, and high alpine ecosystems. Here, we investigated the immunogenetic variation of wolverines in Canada as a surrogate for identifying local adaptation by contrasting the genetic structure at MHC relative to the structure at 11 neutral microsatellites to account for gene flow and drift. Evidence of historical positive selection was detected at MHC using maximum likelihood codon-based methods. Bayesian and multivariate cluster analyses revealed weaker population genetic differentiation at MHC relative to the increasing microsatellite genetic structure towards the eastern wolverine distribution. Mantel correlations of MHC against geographical distances showed no pattern of isolation by distance (IBD: r = -0.03, *p* = 0.9), whereas for microsatellites we found a relatively strong and significant IBD (r = 0.54, *p* = 0.01). Moreover, we found a significant correlation between microsatellite allelic richness and the mean number of MHC alleles, but we did not observe low MHC diversity in small populations. Overall these results suggest that MHC polymorphism has been influenced primarily by balancing selection and to a lesser extent by neutral processes such as genetic drift, with no clear evidence for local adaptation. This study contributes to our understanding of how vulnerable populations of wolverines may respond to selective pressures across their range.

## Introduction

Species are exposed to arrays of selective pressures that often vary spatially and temporally across their range to which they must adapt. The ability of populations to locally adapt to their environments depends on the relative influences of natural selection, gene flow, and drift [1–3]. With many natural populations exposed to unprecedented rates of environmental change such as climate change, anthropogenic modified landscapes, and emerging infectious diseases, understanding patterns of local adaptation provides insight into the capacity for populations to respond to these rapid changes or become extirpated [1]. Northern hemispheres are experiencing accelerated rates of environmental change, with warming temperatures and increasing levels of precipitation [4]. One expected consequence is a significant increase in the emergence of infectious diseases by the northern expansion of disease vectors and their hosts into regions that were previously inhospitable to them [4–6]. It is unclear if northern species have the capacity to adapt to these rapid changes [4,5,7].

Major histocompatibility complex (MHC) genes are the most polymorphic coding regions in vertebrates, and play a crucial function in the adaptive immune response [8]. MHC genes through peptide-binding sites (PBR) are responsible of antigen recognition [9]. The MHC complex is classified in two types: class I genes are associated with intracellular pathogen defence, and class II genes, which are involved with extracellular pathogen and parasite defense [10]. The function of MHC genes is well characterized, and their genetic polymorphism is hypothesized to shift under varying selective pressures [11]. The polymorphism of MHC class II genes have been studied more frequently than class I genes in empirical population genetic studies, and have proven to be an effective genetic marker to test local adaptation across heterogeneous environments (e.g. Atlantic salmon [12], great snipe [13], house sparrow [14], raccoons [15]), as well as a good indicator of wildlife health (e.g. Tasmanian devil [16], grey seals [17]).

High levels of MHC polymorphism are assumed to be maintained by different, but not mutually exclusive mechanisms of balancing selection mediated by pathogen resistance that include heterozygote advantage, negative frequency-dependent selection, and fluctuating selection (reviewed in [9,11,18]). Sexual selection through MHC-based mate choice of pathogen-resistance alleles (influencing offspring fitness) may also explain high levels of MHC polymorphism (e.g., [19,20]). However, like any other genomic region, the spatial and temporal distribution of MHC polymorphism can be influenced by other evolutionary forces such as gene flow and genetic drift [9]. Gene flow can promote the spread of adaptive or maladapted alleles across the landscape, and can offset local adaptation by introducing novel alleles not adapted to the present pathogen pool in a population [21,22]. Alternatively, in species that have small populations, such as species of conservation concern, genetic drift may be a stronger force than both natural selection and gene flow, undermining local adaptation through the erosion of MHC diversity [23,24].

Interactions among pathogens, hosts, and the environment, are highly dynamic processes [25] and the relative influence of selection, gene flow and genetic drift on MHC polymorphism can shift over temporal and spatial scales, or act synergistically [26]. One way to understand the relative influence of gene flow, genetic drift, and selection on spatial patterns of MHC structure is to compare the population genetic structure of this functional molecular marker to that of neutral loci where patterns of genetic structure are influenced by gene flow and drift [11]. For instance, under balancing selection, population differentiation at MHC genes is expected to be weaker relative to differentiation at neutral loci as balancing selection would prevent the loss of rare alleles by drift despite restricted gene flow [18,27]. On the other hand, fluctuating selective pressures, such as from varying pathogen pools across environments, should lead to a decrease of MHC diversity within a population, while increasing MHC genetic differentiation among populations relative to neutral loci (e.g., [12–14,28], but see [29]).

Wolverines (*Gulo gulo*) are mid-size carnivores of high dispersal ability, inhabiting northern ecosystems with a Holarctic distribution. Direct persecution, habitat loss and degradation have negatively impacted this species [30]. Wolverine populations are of varying levels of conservation concern across much of their contemporary range [31]. In North America, wolverine distribution has been reduced by approximately 37% [32], and in some regions of Canada, wolverines have been functionally extirpated (Quebec and Labrador) or persist at low densities (Ontario) [30]. Exposure to reduced pathogen spectrums in northern environments compared to tropical areas [33] is assumed to result in low MHC diversity and limited capacity to resist a wide range of pathogens in northern wildlife (e.g., North American moose [34], polar bears [35], but see bison [36], caribou [37]). However, the opportunistic scavenger behaviour of wolverines may expose them to a variety of pathogens from feeding on animal carcasses [38] across their heterogeneous geographic range covering varied ecoregions from arctic, taiga, mountain cordillera, and boreal forest. Hence, varying selective pressures across the extensive range of wolverines may importantly influence the distribution of MHC polymorphism across populations. Neutral genetic studies for wolverines suggest that female philopatry most likely accounts for the geographical genetic structure of this species. Mitochondrial (mtDNA) genetic structure was much stronger [39–41] relative to the genetic structure observed for neutral microsatellite loci across a broad geographic range, reflecting the long distance dispersal capacity for males [39–44]. Both mtDNA and microsatellites showed higher genetic structure towards the eastern and southern peripheries of the wolverine’s distribution in North America [41,42], which is hypothesized to reflect a historical colonization incursion from west to east during the Holocene [45].

Here, we investigated the spatial distribution of genetic variation of the MHC DRB exon 2 as a surrogate for identifying patterns of local adaptation across the widespread distribution of wolverines in Canada. This was accomplished by contrasting spatial patterns of MHC and neutral microsatellite markers to account for demographic processes on MHC variation, while using wolverines from a region in eastern Russia as a reference point for comparisons. We expected to find higher diversity and similar MHC variation in the core of the wolverine distribution as a matter of extensive gene flow [40,43], with stronger MHC structure towards the eastern distribution from the combination of limited gene flow and increased drift in the smaller eastern populations [41,42,44]. Further, we expected the varying spatial MHC structure to be stronger than the structure from microsatellite loci if fluctuating selection played a major role shaping the distribution of MHC variation (as in other studies e.g., [7–10]). Alternatively, spatially unstructured MHC variation relative to microsatellite loci may reflect the effects of balancing selection (as seen in other species, e g., [27,46]). Investigating how selection and demographic processes shape standing patterns of adaptive genetic variation in wolverines has the potential to improve our understanding of the evolutionary potential of northern wildlife exposed to ongoing and accelerating environmental changes [3, 5, 7].

## Materials and Methods

### Study species and habitat

Wolverines are wide-ranging, occurring in several ecoregions in Canada that are defined by regional climatic, physiographic, and biotic characteristics. Wolverines are well adapted to winter conditions and require presence of snow (depth 1m) for their dens [30]. Nunavut (NU) is the northernmost population in our study, and belongs to the Arctic ecoregion, the coldest and driest region in Canada, where permafrost extends across vast areas and vegetation is scarce. Wolverine distribution in areas of Yukon (YK) and Northwest Territories (NWT) corresponds to the Taiga ecoregion, which is characterized by coniferous evergreen forest, cool summers and long, cold winters with high influence of arctic air [47]. At the southwest of YK and in most of British Columbia (BC), the occurrence of high elevation mountainous ranges creates a complex variety of climatic and topographic conditions, where vegetation varies from alpine tundra and dense coniferous forest to sagebrush and grasslands [47]. Wolverines in the Boreal Plains (in Alberta (AB) and Saskatchewan (SK)) and the Boreal Shield (SK, Manitoba (MB), and Ontario (ON)) persist at lower densities [30]. These areas are the largest extension of flat land in Canada, where deciduous trees are more common. Continental and maritime influences provide milder winters and warmer summers relative to the western ecoregions of the wolverine distribution [47].

### Sampling and microsatellite loci

Samples used in this study were a subset of those previously analyzed by Kyle & Strobeck [42,43] and Zigouris et al. [41] using neutral microsatellite loci and mitochondrial DNA control region. We selected a total of 269 individuals from nine regions: eastern Russia (RU), NU, YK, NWT, BC, AB, SK, western MB and western ON. Bone and earplug samples for RU, YK, and NU were provided by the University of Alaska, Fairbanks Museum, while pelt and earplug samples for AB, BC, NWT, SK, MB and ON were obtained through fur auction houses, pelt dealers, hair snares or incidental deaths. Sampling protocols were approved by the Ministry of Natural Resources of Canada, and all samples were collected post 1990 and stored at -80°C for their long-term preservation. Microsatellite data for eleven loci (Tt1, Tt4, Gg-3, Gg-4, Gg-7, Gg-14, [48]; Ggu-101, Ggu-216, Ggu-234; [49]; Mvis-75 [50], Lut-604 [51]) came from Kyle & Strobeck[42,43] and Zigouris et al. [41]. Genotypes used in this study have previously been confirmed to correspond to unique individuals by assessing the genotype matches among samples and obtaining consensus genotypes (see Zigouris et al [37]).

### MHC DRB-2 profiling

Oomen et al. [52] previously characterized MHC DRB exon 2, including PBR sites, in a subset of wolverines by contrasting two protocols, 454 pyrosequencing, and cloning and Sanger sequencing, which identified the presence of 10 MHC alleles. To test our research predictions, we screened a larger number of samples and populations for the DRB exon 2 using the 454 pyrosequencing protocol described in Oomen et al. [52]. In brief, total genomic, good quality DNA was extracted using the QIAGEN DNeasy blood & tissue kit according to the manufacturer’s instructions. DNA was quantified using PicoGreen^®^ (Invitrogen, Burlington, Canada) and standardized to 2.5 ng/μl. Amplicon libraries of a 185-bp fragment of the MHC class II DRB exon 2 were amplified using a modified reverse primer DRB-3c (CCGCTGCACAGTGAAACTCTC; [53]) with a MID adaptor (MID1- MID6, MID11; Roche Diagnostics), and modified forward DRB-5c primer (TCAATGGGACGGAGCGGGTGC) with a MID adaptor (MID1-MID8, MID10-MID11, MID13-MID16; Roche Diagnostics). MIDs are sequence tags for individual identification, and the combinations of these 14 MIDs resulted in 96 unique individual tags that differed by at least 6-10bp, which makes misassignment of reads to individuals due to sequencing errors completely unlikely. Amplicon libraries were quantified using PicoGreen^®^ and pooled in equimolar ratios to reduce sequencing bias among amplicons. Pooled libraries from 70–90 individuals were prepared for 454-sequencing using a Roche GS Junior System. To verify the consistency of the MHC profiling we ran 42 individuals in duplicate on different 454 sequencing runs.

Data generated from the 454 sequencing can be challenging to analyze as it includes spurious DNA sequences generated during the PCR or 454 sequencing, which can be confounded with true MHC variants, particularly in species that present large differences in the number of MHC loci [54]. We expected the number of MHC DRB-2 variants to be low in wolverines as only 10 alleles had been identified previously [52]. We used these characterized MHC alleles as a baseline to validate the MHC individual genotyping, and following the multi-step criteria described in Sepil et al. [55] to filter any additional true MHC alleles from spurious DNA sequences. Raw reads in FASTA format were used as input into the jMHC software [56], which extract reads (i.e., variants) that include complete primers and tags and assigns those reads to their corresponding individual. Sequences lacking complete primers and tags, with ambiguous base pairs, containing indels, or sequences that did not match the expected allele size of 185 bp were discarded. We calculated the maximum per amplicon frequency (MPAF) for each variant, which is the maximum proportion of the individual’s reads for a given variant among all individuals in which the variant was present. The data set comprised 85 potential allele variants with a frequency distribution ranging from 0.1 to 54%. Oomen et al. [45] determined that true MHC alleles occurred within a MPAF threshold of 4–6%. Based on the known 10 MHC alleles, we observed that the previously characterized 10 MHC alleles could be present within an individual with a minimum MPAF frequency of 4%. The minimum number of reads required for reliable genotyping was found to be ≥150, which was determined using duplicated samples with large variations in the total number of reads (e.g., min = 93, max = 2069 reads), but which matched completely in their MHC profiling. Variants within the range of 0.1 to 4% were compared against the true alleles to check if their sequence variation could be explained by a difference of 1-2bp from a parental true allele present in the individual, or contained premature stop codons, or produced a frame-shift mutation. We removed variants that were present only in one individual or that could not be verified in duplicated samples.

### Data analyses

#### Microsatellite loci

Departures from Hardy-Weinberg equilibrium (HWE) and linkage disequilibrium (LD) for each location at the 11 microsatellite loci were tested using FSTAT v2.9 [57]. Observed (*H*_*o*_) and expected heterozygosity (*H*_e_) were estimated in FSTAT v2.9. Rarefied allelic richness (A*r*) corrected for sample size was estimated in HP-RARE [58]. Patterns of genetic structure were analyzed by Bayesian clustering analyses in STRUCTURE v2.3 [59] by varying the likely number of clusters (*k*) from 1 to 10 allowing for genetic admixture, correlated allele frequencies, and with no prior information of populations or sampling locations using 200,000 burn-in steps followed by 400,000 post-burn MCMC iterations. This process was repeated eight times for each value of *k*. The most likely number of *k*-clusters was chosen by compiling runs using STRUCTURE HARVESTER v.0692 [60] and assessing the increase in pr (*X|K*), and using the ad hoc Δ*K* method [61]. Individual membership probabilities of the inferred *k*-clusters from eight independent replicates were averaged using CLUMPP v1.1.2 [62], and clusters were visualized using DISTRUCT v1.1 [63].

#### Tests for selection and recombination at MHC

Oomen et al. [52] previously determined that MHC DRB alleles showed signatures of positive selection based on the overall Z-test of positive selection, which estimates the ratio of non-synonymous (d_*N*_) to synonymous (d_*S*_) substitutions. We screened for historical positive selection on each codon site based on maximum likelihood methods. Maximum likelihood estimators of ω (ω = d_*N*_ */*d_*S*_ with positive selection indicated by ω = d_*N*_ */*d_*S*_ > 1) among codons were obtained in Codeml in the PAML4 software [64]. We tested six models allowing for different selection intensity among sites: M0 (one ratio ω), M1a (nearly neutral), M2a (positive selection), M3 (discrete), M7 (nearly neutral with beta distribution approximating ω variation) and M8 (positive selection with beta distribution approximating ω variation) [65,66]. We used likelihood ratio tests (LRTs) to determine if models including positive selection (M3, M2a and M8) resulted in the best fit to our data by comparing three nested models: M0 vs. M3, M1a vs. M2a, and M7 vs. M8. Positively selected sites were identified by Bayes empirical Bayes procedure (BEB) for models M2a and M8 [67]. We also tested for codon based positive selection using the fixed effects likelihood (FEL) and mixed effects model of evolution (MEME) implemented in the HyPhy software (hosted at Datamonkey: http://www.datamonkey.org/ [68–70]). We checked for signatures of recombination using the genetic algorithm recombination detection (GARD) method using the Datamonkey website.

#### MHC DRB-2

Variation in the number of MHC loci within individuals is a common feature in many vertebrate species e.g., [15,54,55,71,72] that is the result of gene evolution by a birth and death process, where some duplicated genes are maintained by balancing selection for a long time, whereas others are eliminated or become non-functional [73]. This MHC feature makes the assignment of detected co-amplifying alleles to specific loci challenging [54]. In wolverines, we found up to five alleles per individual, which suggest the presence of at least three DRB loci. We could not ascribe alleles to loci and thus we estimated MHC diversity using different approaches. First, at the population level, we calculated average nucleotide diversity (π) for each sampled location in ARLEQUIN v3.11 [74] by entering the MHC sequence data and their respective haplotype frequency for each sampling location. Similar to Ekblom et al. [13], we calculated MHC relative allele frequencies by counting the number of individuals carrying a particular allele divided by the total number of alleles per sampling region. Additionally, we used measures independent of allele frequency, including the total number of alleles [75,76], and MHC-like genotype diversity (GT) per population. GT was estimated by identifying unique allele combinations within individuals. We used multilocus matches in GENALEX v6.5 [77] to detect unique MHC genotypes based on a binary-coded data. Lastly per individual, we used the mean number of alleles [75] and an index of allele diversity, which was calculated by counting the number of alleles per individual and dividing by the maximum number of alleles found within individuals in the total data set. We used this index to facilitate comparisons among populations because it can range between 0.4 (minimum of 2 alleles) to a maximum value of 1 (5 alleles).

#### Comparisons among markers

We estimated genetic differentiation at MHC and microsatellites through pairwise F_*ST*_ distances in ARLEQUIN v3.11 [74]. F_*ST*_ between all pairs of populations was computed for the MHC sequence data using the Jukes-Cantor distance model [78] as the best nucleotide substitution model that fit our MHC data estimated in MEGA v6 [79]. F_*ST*_ for microsatellite loci was calculated using the number of different alleles [80]. Statistical significance of F_*ST*_ values between all pairs was estimated by 1000 randomizations. In addition to F_*ST*,_ we estimated Jost’s D actual differentiation estimator D_*EST*,_ which partitions diversity into independent within and between subpopulation components [81], and has been suggested to better describe genetic differentiation when within-population genetic diversity is high [82]. D_*EST*_ was calculated in SPADE [83]. To assess the degree of genetic structuring among regions for MHC and microsatellite loci, we performed an analysis of molecular variance (AMOVA). AMOVA was calculated by partitioning the genetic variance among the nine sampling regions and by using the MHC allele nucleotide sequences as haplotypes and their frequencies per region, while for microsatellite we used the co-dominant genotype data. Significance of AMOVA components were tested with 10000 permutations using ARLEQUIN v3.11 [74].

In an attempt to make the genetic structure analyses as comparable between markers as possible, we used binary-encoded data with each allele considered a separate dominant locus (presence 1/absent 0) for microsatellites and MHC (as per [26,71]). Hence, with the MHC and microsatellite binary-encoded data, we performed AMOVA, and clustering analysis for dominant markers using STRUCTURE to identify the most likely number of *k*-clusters. We ran STRUCTURE using genetic admixture, correlated allele frequencies, and no prior population location information. For each *k*, from 1 to 10, we performed 5 independent runs of 200,000 burn-in steps followed by 400,000 post-burn MCMC iterations. Comparing population genetic differentiation between MHC and microsatellite loci can be challenging because these markers differ in their mutational, HWE, and LD population equilibrium assumptions. We took the approach of Lamaze et al. [84] to directly contrast the degree of population genetic differentiation at microsatellites and MHC and evaluate the influence of neutral processes shaping MHC population structure. We performed a co-inertia analysis (CoA), which is a multivariate method that identifies joint trends between two data sets containing the same observations (e.g., same individuals) [85]. This method provides advantages over traditional genetic differentiation estimates such as *F*_*ST*_ because comparisons are not limited to population pairs. CoA does not rely on mutational and equilibrium assumptions, and genetic variation is maximized among population groups using between-class principal component analyses (PCA) as input in CoA [86]. CoA describes the common structure between data sets, and allows for a visual assessment of the co-relationship of microsatellites and MHC among and within populations. CoA has been deemed useful in assessing the genetic co-structure between MHC and microsatellites and infer patterns of local adaptation [84]. We calculated genetic distances for the MHC and microsatellites binary-encoded data using the Jaccard similarity coefficient (S3 coefficient [87]). For each distance matrix, we performed a between-class PCA using populations as predefined groups; subsequently these principle components were input for CoA using the *ade4* R package [88]. The first two axes of the CoA plot contain the maximum squared covariance between data sets, where each population is represented with a vector (arrow): the tip of the arrow shows the position of the MHC and the start (the dot) refers to the position of the microsatellites on the factorial map. The length of the vector is inversely proportional to the co-variation between MHC and microsatellite data sets. If both genetic markers have strong joint trends, the arrow would be short, while large when weak. The global significance of the co-relationship between MHC and microsatellite was tested using 1000 bootstraps.

To account for the potential effect of restricted gene flow on MHC population genetic differentiation, we examined if population differentiation across the wolverine Canadian distribution followed an isolation by distance (IBD) model. We used simple and partial Mantel correlations to assess the significant relationship between geographical distances and population genetic distances (F_ST_ and D_EST_) for MHC and microsatellite loci. As we were interested in detecting genetic differentiation from the wolverine’s Canadian range we excluded samples from RU in IBD tests given their physical geographical separation. Partial Mantel correlations were used to test the effect of geographical distance on MHC genetic distances, while controlling for the genetic differentiation at neutral microsatellite loci. Log-transformations of geographic distances (km) were performed to improve linearity for the Mantel test. We also tested for correlations between MHC and microsatellite pairwise F_ST_ and D_*EST*_ distances. Significance of Mantel correlation coefficients were tested by permuting observations 1000 times using the R library *vegan* [89]. Lastly, we assessed the relationship between microsatellite (Ar) and MHC (mean number of alleles) diversity using a Pearson product moment correlation test.

## Results

The mean coverage for MHC DBR exon 2 was 1,412 reads (SD ± 1,387) per individual. We discarded 32 individual samples that had a low number of reads (mean = 42.8). We did not observe a significant correlation between the number of alleles and coverage (r = 0.04, *P* = 0.51), which indicates that our coverage was sufficient for reliable genotyping and that genotyping bias was negligible. From 42 samples run in duplicate across independent runs, 37 samples produced a complete match of MHC allele profiles (88% repeatability rate, which is similar to other reported studies [54]), and five samples (12%) resulted in partial allele matches of the up to 5 alleles/individual. No samples were found to provide zero agreement among the allele calls. We found the same ten MHC alleles that were previously identified and validated in Oomen et al. [52] (GenBank accession numbers JX409655–JX409665). We found an additional three variants that were present only in one individual and with low number of reads (MPAF < 4%). These variants were not included in subsequent analysis as we were unable to confirm them as true alleles due to low frequency.

### MHC selection and recombination tests

Evidence of historical positive selection was found for all models with selection M2a, M3, and M8 in Codeml (Table 1). Based on LRTs, these models had a better fit to the data relative to models without selection (Table 2). For the M2a and M8 models, five codons were identified under positive selection, whereas the M3 model identified 8 codons (Table 2). REL identified one codon as positively selected (site 68, *P* < 0.05) and MEME identified two codons (sites 39, and 56, *P* <0.05). These sites were in agreement with the codons identified in Codeml, and all codons were peptide-binding regions (PBR) [52]. Using the GARD recombination algorithm, only one out of 25 potential breakpoints was significant for recombination (site 28, *P* <0.0001). This site was located in proximity to one codon detected under selection (site 29, Table 2).

**Table 1:** Results from maximum likelihood codon-based models of selection using Codeml.

Model	P	ln *L*	Parameter estimates	Positively selected sites
M0 (one ratio)	1	-537.57	*K* = 1.51, ω = 1.174	None
M1a (nearly neutral)	2	-527.69	*K* = 1.126, *p*_0_ = 0.678, *p*_1_ = 0.322, ω0 = 0, ω1 = 1	Not allowed
M2a (positive selection)	4	-516.17	*K* = 1.569, *p*_0_ = 0.668, *p*_1_ = 0.185, *p*_2_ = 0.147, ω_0_ = 0, ω1 = 1, ω_2_ = 8.99	*12Y*, **39D**, **56N, 60Y**, *68G*
M3 (discrete)	5	-516.13	*K* = 1.627, p0 = 0.795, p1 = 0.186, p2 = 0.019, ω_0_ = 0.043, ω_1_ = 6.268, ω_2_ = 19.897	**10D, 12Y, 13F, 29Y, 39D, 56N, 60Y, 68G**
M7 (beta)	2	-529.17	*K* = 1.23, p = 0.005, q = 0.005	Not allowed
M8 (beta and omega)	4	-516.17	*K* = 1.567, *p*_0_ = 0.849, *p*_1_ = 0.150, p = 0.0277, q = 0.0277, ω = 8.813	*12Y*, **39D**, **56N, 60Y, 68G**

P = number of parameters in the ω distribution; *K* = estimated transition/transversion rate; ω = selection parameter; p_n_ = proportion of sites that fall into the ω_n_ site class; p, q = shape parameters of the β function (for models M7 and M8). Positively selected sites denoted in cursives were significant at *P* > 95%, while bold sites were significant at *P* > 99%.

**Table 2:** Goodness of fit based on likelihood ratio test for three nested models of codon evolution. LRT statistic was computed using 2(Ln _mod1_-Ln _mod2_) where Ln represents the likelihood of the two compared models (mod1 and mod2).

Model compared	LRT statistic	d.f.	Significance
M0 vs. M3	42.87	4	*P* < 0.0001
M1a vs. M2a	23.04	2	*P* < 0.0001
M7 vs. M8	25.99	2	*P* < 0.0001

d.f. degree of freedom.

### Genetic diversity: microsatellites and MHC

For the 11 microsatellites, there were no significant departures from HWE and LD within regions after Bonferroni correction. MB and ON showed the largest values of expected heterozygosity and allelic richness for microsatellites, while AB and BC showed the lowest heterozygosity (Table 3). For the MHC data, a large proportion of individuals presented four alleles (39.9%) followed by individuals with three alleles (30.4%) and two alleles (26.7%). A few individuals (2.9%) from RU, NWT, MB, and AB had five alleles (S1 Fig). The average number of MHC alleles per individual within regions ranged from 2.9 ± 0.95 (YK and SK) to 3.6 ± 0.77 (MB), but there were no significant differences among regions (*P* < 0.05). We identified 46 MHC-like genotypes for the complete data set based on the unique allele combinations within individuals. Within sampling locations, NU and BC showed the largest number of MHC-like genotypes (GT = 20), whereas YK the lowest (GT = 9). However, RU showed the largest number of unique MHC-like genotypes (P_GT_ = 4; Table 3). For the MHC individual allele diversity index (A), MB showed the highest value (A = 0.6), while SK and YK the lowest (A = 0.49). There was a significant correlation between the mean number of MHC alleles per individual and microsatellite allelic richness (r = 0.76, *P* = 0.02).

**Table 3:** Estimates of genetic diversity for eleven neutral microsatellite loci and MHC DRB-2 across nine sampling regions for wolverines.

		Microsatellites			MHC				
Region	Samples	*H*_*o*_	*H*_*e*_	*A*_*r*_	H	π	GT	P_GT_	*A*
RU	26	0.55	0.65	4.02	8	0.052	12	4	0.54
YK	16	0.66	0.67	3.98	8	0.048	9	1	0.49
NWT	35	0.63	0.64	4.08	9	0.042	14	2	0.54
NU	56	0.65	0.65	4.04	10	0.041	20	1	0.53
BC	41	0.58	0.61	3.95	9	0.051	20	3	0.51
AB	19	0.57	0.60	3.99	9	0.052	13	2	0.55
SK	13	0.65	0.62	3.84	7	0.046	7	0	0.49
MB	28	0.60	0.68	4.14	8	0.052	17	0	0.60
ON	35	0.68	0.68	4.07	8	0.05	13	0	0.53

Abbreviations as follows: Observed heterozygosity (*H*_*o*_), expected heterozygosity (*H*_*e*_), rarified allelic richness (*A*_*r*_). Number of MHC alleles (H), nucleotide diversity (π), number of MHC-like genotypes (GT), private number of MHC- like genotypes (P_GT_), MHC individual allele diversity (*A*).

### Population differentiation: microsatellites and MHC

**Fig 1:**
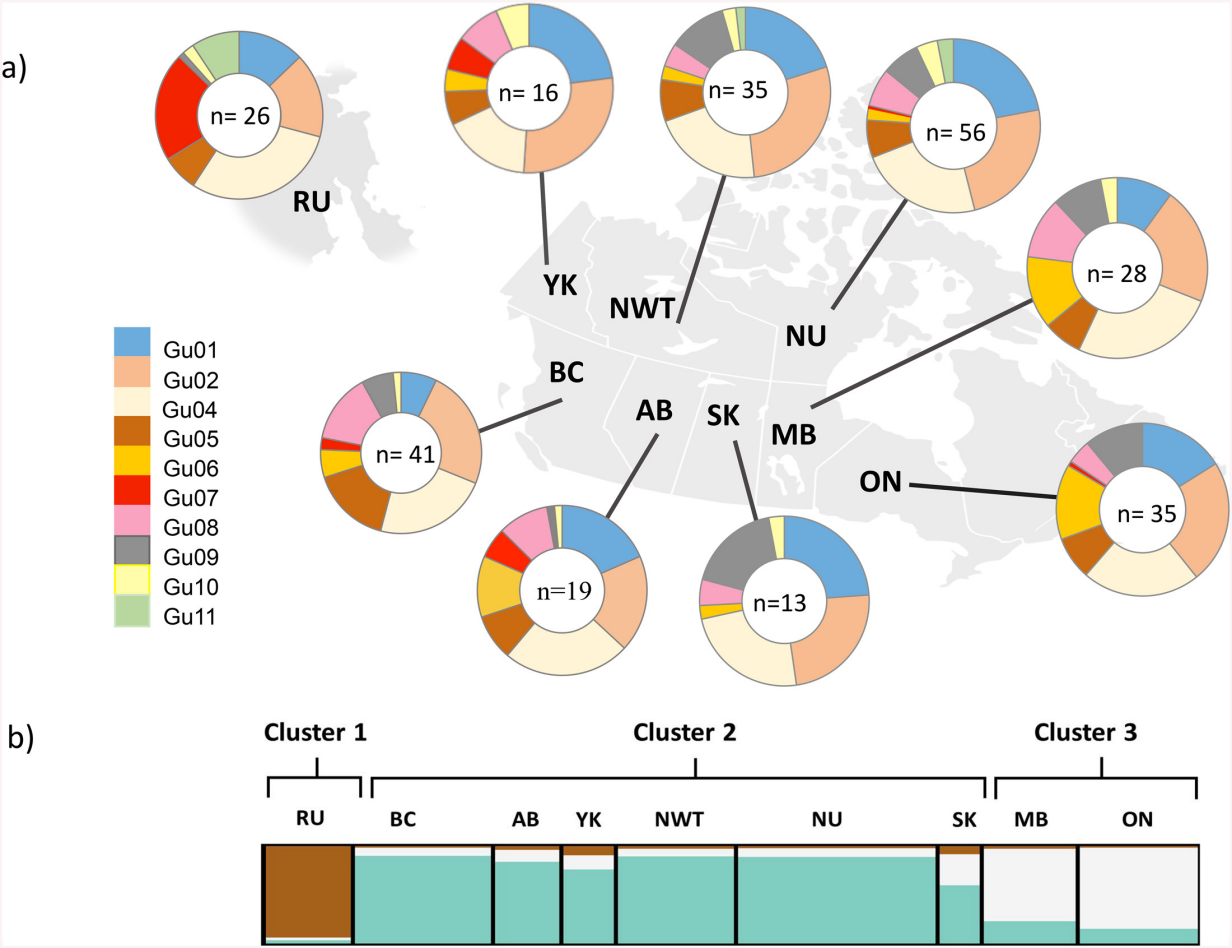
Patterns of microsatellite and MHC genetic variation within nine sampled regions. (a) Relative frequency distribution of ten MHC alleles per sampled region. Each color of the pie chart represents an MHC allele, while its size is proportional to the frequency of that allele within a location. Numbers within pie charts denote sample size. (b) STRUCTURE barplot of population membership scores for inferred *k* = 3 genetic clusters for 11 microsatellites.

Relative frequency distributions of MHC alleles within sampling regions showed three alleles (Gu01, Gu02, Gu04) with the highest frequencies (summing up to 60%-70%; Fig 1a). Allele Gu11 was present only in RU, NWT, and NU. Allele Gu07 was present in low frequencies in all regions, except in RU that had the highest Gu07 frequency, and alleles Gu06 and G08 were exclusive to Canada (Fig 1a).

Bayesian cluster analysis in STRUTURE using the MHC binary-encoded data did not detect any genetic cluster exclusive to a sampling population (S2 Fig). In contrast, for the microsatellite co-dominant data, STRUCTURE identified geographical structuring in three genetic clusters as determined by the *ΔK* plot that peaked at *k* = 3. Cluster 1 distinguished samples from RU, cluster 2 pooled samples from the central part of the wolverine distribution, YK, BC, AB, NWT, NU and SK, while MB and ON formed a third genetic cluster (Fig 1b). These three clusters were also identified with the binary-encoded microsatellite data (S2 Fig).

**Fig 2:**
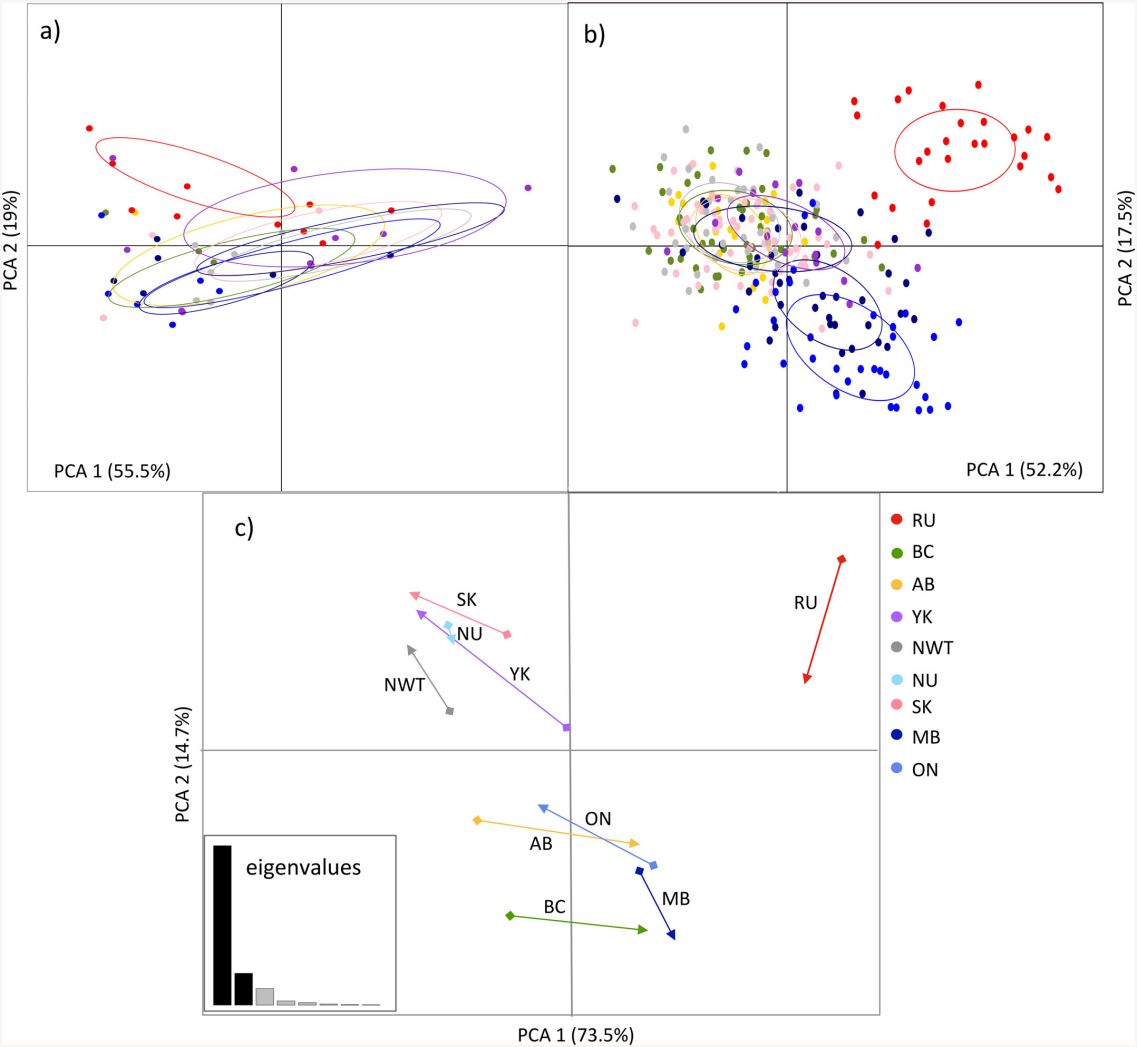
Co-inertia analysis (CoA) between MHC and microsatellite binary-encoded data for nine regions. Ordination of the first two between-class axes for (a) MHC and (b) microsatellite loci, where dots represent individuals constrained by sampling locations distinguished in different colors; (c) CoA plot, showing the relative position of each population on the factorial plane for the first two CoA eigenvalues and given by the co-variation between MHC and microsatellite data seta. The dots represent the variation observed at microsatellites, while the arrows represent the variation at MHC. The length and direction of the vector denote the translational coefficient of the population position relative to each other, while the strength of the correlation between microsatellite and MHC data sets for each population is inversely correlated with the vector length. Inset figure shows CoA eigenvalues, where each bar represents the proportion of inertia contained for each eigenvalue.

The AMOVA of the microsatellite data showed a much higher proportion of the genetic variance explained among populations (7.04%) relative to MHC (0.98%), but both Φ_ST_ were statistically significant (*P* < 0.05). AMOVAs using binary-encoded data for both markers showed the same trend, but the difference between markers was much smaller as the proportion of the genetic variance explained for microsatellites was 14%, while 10% for MHC. Between-class PCA axes for the MHC showed no clear differentiation of sampling locations as there was large overlap of individuals among populations (Fig 2a). For microsatellites, the first two PCA axes separated RU samples, while the second axis separated MB and ON from the rest (Fig 2b). This PCA group scattering was similar to the three STRUCTURE clusters. The CoA plot showed the co-structure between the MHC and microsatellites (Fig 2c). RU was discriminated along the first axis, which accounted for the large portion of the variance (76%), while the rest of the populations split in two groups along the second axis. The co-variation within populations showed that NU had the shortest vector length and thus the highest co-relationship between MHC and microsatellites, while YK, AB, RU showed the lowest (i.e., larger vector, Fig 2c). There was no significant global correlation between the MHC and microsatellites (RV = 0.51, *P* = 0.09).

Pairwise *F*_ST_ values were higher for microsatellites, ranging from 0.005 to 0.17, than for MHC, where Φ_ST_ ranged from -0.008 to 0.04 (Table 4). Almost all *F*_*ST*_ pairwise comparisons were statistically significant for microsatellites (33 out of 36), while only 9 out of 36 Φ_ST_ comparisons were significant for MHC, and mainly for pairwise comparisons that included RU followed by NU (Table 4).

**Table 4:** Population pairwise F_*ST*_ values for eleven microsatellite loci (above the diagonal) and for MHC DRB-2 (below the diagonal) in nine sampling regions for wolverines. Values in bold indicates statistically significance after 1000 permutations.

	RU	YK	NWT	NU	BC	SK	AB	MB	ON
RU		**0.110**	**0.132**	**0.147**	**0.166**	**0.139**	**0.138**	**0.138**	**0.155**
YK	0.022		**0.022**	**0.041**	**0.040**	**0.068**	0.014	**0.030**	**0.061**
NWT	**0.040**	0.000		**0.015**	**0.030**	0.013	0.005	**0.048**	**0.080**
NU	**0.033**	0.007	-0.004		**0.055**	**0.032**	**0.029**	**0.056**	**0.087**
BC	0.017	-0.005	0.009	**0.014**		**0.068**	**0.013**	**0.065**	**0.106**
SK	0.026	0.001	-0.007	-0.001	-0.003		**0.026**	**0.054**	**0.088**
AB	0.006	-0.008	**0.020**	**0.022**	-0.005	0.010		**0.044**	**0.079**
MB	**0.022**	-0.002	**0.021**	**0.027**	-0.004	0.001	-0.006		**0.020**
ON	**0.019**	-0.010	0.009	**0.015**	-0.005	0.001	-0.008	-0.005	

Most D_*EST*_ distances were higher for microsatellites than for MHC (S1 Table). Both F_*ST*_ and D_*EST*_ values consistently placed RU as the region most differentiated. Excluding RU from the Mantel test, there was a non-significant correlation of MHC F_*ST*_ or D_*EST*_ distances and geographical distance (F_ST_: M_*r*_ = -0.03, *P* = 0.9, D_*EST*:_ M_*r*_ = 0.1, *P* = 0.6). In contrast, for microsatellites there was a strong and significant correlation of F_*ST*_ (M_*r*_ = 0.54, *P* = 0.01) and D_*EST*_ (M_*r*_ = 0.46, *P* = 0.03) distances with geographical distance. Controlling for the effect of neutral genetic differentiation on MHC F_*ST*_ or D_*EST*_ and geographical distances in partial Mantel test did not change observed trends (F_ST_: M_*r*_ = -0.04, *P* = 0.5, D_EST_: M_*r*_ = 0.13, *P* = 0.3). There was a non-significant correlation between pairwise *F*_*ST*_ distances of MHC and microsatellites (M_*r*_ = 0.09 *P* = 0.4) or D_EST_ distances (M_*r*_ = -0.03 *P* = 0.5).

## Discussion

Under balancing selection, genetic structuring at MHC loci is expected to be low because MHC polymorphism would be maintained across populations in the long term, even in the event of restricted gene flow [18,27]. In this study, by comparing patterns of genetic structure of MHC and neutral microsatellites across a broad geographic distribution of wolverines in Canada, we suggest that MHC genetic variation has primarily been influenced by balancing selection and to a lesser extent by neutral processes, with no evidence of local adaptation. Our conclusion is supported by several lines of evidence that showed weaker patterns of genetic structuring for MHC relative to microsatellite loci. Specifically, (*i*) Cluster analyses revealed no structure at MHC, whereas genetic structuring was observed towards the eastern extent of the Canadian wolverine distribution for microsatellites. This observation was in agreement with results from (*ii*) AMOVAs, which showed a larger proportion of genetic variance explained for microsatellites than for MHC. Remarkably, (*iii*) only 25% of pairwise Φ_*ST*_ comparisons for MHC were significant, while F_*ST*_ values for microsatellites were higher and significant in most cases (92%). We found (*iv*) no evidence of isolation by geographical distance at the MHC, whereas a strong and significant pattern of isolation by distance was observed for neutral microsatellite loci. Moreover, (*v)* the comparison of MHC and microsatellite data using CoA revealed a non-significant global correlation between markers.

Evidence of historical selection at MHC was supported by all maximum likelihood codon-based selection models (M2a, M3, M8), which produced a ‘best fit’ to the data compared to models without selection. Importantly, all codons detected under positive selection were PBR sites [52], which are involved in pathogen binding recognition [9]. Recombination also was detected at one site near a PBR codon. Recombination, gene duplications, and point mutations, are common features in the evolution of MHC polymorphism [8,90], and have been reported to occur in several species [91–93].

Consistently, we observed that RU was the region most differentiated at both MHC and microsatellite loci. For example, two MHC alleles that were rare in Canada were in high frequency in RU (alleles Gu07 and Gu11, Fig 1a). RU also showed moderate levels of MHC diversity compared to other sampled regions, but RU had the largest number of unique MHC-like genotypes. Wolverines in RU were historically connected to North America through the Bering Strait during past glaciations, but present-day populations from eastern and western hemispheres are not thought to have intermixed since the glacial retreat of the Holocene [94]. Restricted gene flow would be expected to increase the strength of local adaptation at functional MHC by selective pressures such as from local pathogen pools [95], however, the observed higher genetic differentiation at neutral loci relative to MHC may indicate that drift rather than local adaptation may explain the differentiation observed for RU at MHC [45].

Genetic studies using mtDNA control region have shown increasing genetic structuring towards the eastern range of wolverines, likely as the result of a historical colonization incursion from the Bering Strait to North America during the Holocene [38, 41]. Microsatellite data revealed lower genetic structure across much of the wolverine range relative to mtDNA, which suggests contemporary long distance dispersal [40–43]. Given the potential of longstanding reduced gene flow at the eastern periphery of the wolverine distribution (MB and ON), we expected to find stronger MHC differentiation as the result of diversifying selection on MHC [14,96]. However, we found similar MHC variation of peripheral populations relative to other populations. MHC loci under balancing selection (likely from mechanisms of heterozygote advantage or negative frequency dependent selection) are expected to show lower genetic differentiation than neutral loci because advantageous alleles would have higher effective migration rates than neutral loci [97]. Consistently among analyses (e.g., AMOVA, STRUCTURE, and PCAs), we found that MHC showed weaker structuring relative to the genetic structuring shown by microsatellites, a pattern that remained when both markers were binary-encoded for analyses. While there may be limitations to uncover patterns of genetic structure when using binary-encoded data, this approach does provide a mechanism to compare multilocus MHC and microsatellite data[26,71]. One limitation that was noted using the binary-encoded data was in AMOVAs that showed a smaller difference between markers in the proportion of the genetic variance explained among populations. This difference might reflect the limitations in detecting genetic structure when transforming to binary-encoded. However, by contrasting the results of the co-dominant and binary-encoded data for microsatellites we observed a consensus of genetic structure patterns from the STRUCTURE and multivariate analyses. Given this agreement, we take these data to suggest that the genetic structure at neutral microsatellites is likely stronger than the structure present at the MHC loci under investigation.

Further, by comparing trends in the degree of genetic differentiation between microsatellite and MHC using CoA, we assumed that if neutral processes have influenced the structure of MHC polymorphism in wolverine populations, then MHC structure should be similar to the genetic structure shown by loci evolving under neutrality. This assumption is based on the fact that gene flow and drift would have lead to similar patterns of genetic differentiation at both neutral and functional loci [9,11,84]. The separation of RU from other regions in CoA was in agreement with the largest differentiation of this population shown by both markers. The CoA separation of the Canadian wolverines in two groups did not correspond to the genetic structuring towards the east observed from microsatellites alone (as in STRUCTURE and PCA). This may indicate the lesser agreement between markers on spatial patterns of genetic differentiation among populations. As well, within populations, we did not observe a strong co-relationship between MHC and neutral loci (except NU that had the shortest vector length), which overall suggest a relatively low influence of neutral processes on MHC differentiation in wolverines across Canada. Weak genetic differentiation in MHC, despite divergence at neutral loci across large geographic scales, has previously been documented [12,71]. This observed pattern has been hypothesized to result from maintenance of ancestral MHC polymorphism [29]. Lower genetic structuring at MHC relative to neutral loci has been observed in several species (e.g., jumping rat [98], island foxes [27], the black grouse [23], zebras [75]), whereas other studies have found higher genetic differentiation in MHC as indicative of local adaptation despite occurrence of gene flow (e.g., Atlantic salmon [12], great snipe [13], house sparrow[14]). The effect of balancing selection on MHC for wolverines may indicate homogeneous selective pressures despite the heterogeneity of habitats along their range. Other studies in cold-adapted species such as the Finnish wolf have found evidence of strong balancing selection at MHC despite restricted gene flow [99].

In terms of genetic diversity, we found a significant correlation between microsatellite allelic richness and the mean number of MHC alleles, which may suggest that neutral processes such as drift have played a role influencing levels of population genetic diversity [9]. Despite an overall loss of MHC alleles in a population by drift, balancing selection through heterozygote advantage is hypothesized to influence levels of MHC diversity within individuals [95,99,100]. MHC heterozygosity has been associated with increased fitness [101,102], which has been found independent of genome wide-heterozygosity [102]. Balancing selection over drift has been documented to maintain MHC diversity in small populations with restricted gene flow [27]. Interestingly, the two small eastern populations of MB and ON did not show reduced genetic diversity, but instead had the highest values of heterozygosity and allelic richness at neutral loci and high MHC individual allele diversity. Although, similar levels of MHC and neutral genetic diversity suggest the action of drift on population genetic diversity [96,86,101], drift may not be strong enough to offset the effect of selection.

Studies have reported numerous associations between levels of MHC diversity [29,102], and pathogen resistance, and probability of survival to adulthood [17]. Specific parasitology studies in wolverines from Alaska, NU, and NWT show high prevalence of parasites such as helminthes [103], roundworms *Trichinella* [104], protozoan *Sarcocystis* [105], and canine viruses (distemper, parvovirus, adenovirus, [106]). Unfortunately, these data do not provide a systematic evaluation of pathogens or pathogen diversity affecting wolverine populations across their range. As such, we could not investigate if individual MHC diversity or specific MHC allele combinations (i.e. genotypes) are associated with differential resistance to specific pathogens and fitness. Parasite diversity in arctic systems, however, is normally considered low [107], and could explain the reduced number of MHC alleles in wolverines relative to other mammals (e.g., raccoons [15], alpine chamois [108]).

## Conclusions

Our results suggest that genetic variation at MHC DRB exon 2 has been influenced primarily by balancing selection and to lower extent by neutral processes, but shows no clear evidence for local adaptation. Overall, this study contributes to our understanding of how vulnerable populations of wolverines may respond to selective pressures across their range. Further research would be necessary to investigate the relationships of MHC diversity and pathogen resistance, in particular towards the eastern wolverine distribution, where densities are low. The analysis of MHC variation provides a good framework to investigate local adaptation and genetic health in wildlife populations [11], but only provides partial understanding of how species adapt to disease [109]. As such genetic investigations in other immunity-associated genes (e.g., [110]) are necessary to provide a more comprehensive understanding of the species genetic potential for adaptation. This is particularly important, in the face of climate change for northern environments, where warming temperatures are predicted to increase the impact of emerging diseases on wildlife [6].

## Supporting Information

S1 Fig Frequency distribution of the number of MHC alleles per individual within nine sampled regions. (TIF)

S2 Fig Patterns of microsatellite and MHC genetic variation using binary-encoded allele data (presence 1/ absence 0) for nine sampled regions: Bar plots of population membership scores for (a) inferred *k = 3* genetic clusters for 11 microsatellites, and (b) for *k* = 2 genetic clusters for MHC as identified in STRUCTURE using 5 independent simulation runs. Although STRUCTURE identified *k* = 2 in the MHC data, there was no evident pattern of population genetic structure shown in the structure bar plot.
(TIF)

S1 Table Population pairwise D_*EST*_ values for eleven microsatellite loci (above the diagonal) and for MHC DRB-2 (below the diagonal) in nine sampling regions for wolverines. (DOC)
