# Supplementary figures and images for: Lack of Spatial Immunogenetic Structure among Wolverine (Gulo gulo) Populations Suggestive of Broad Scale Balancing Selection
Source: PLoS One. 2015 Oct 8;10(10):e0140170. doi: 10.1371/journal.pone.0140170 (PMC4598017; doi:10.1371/journal.pone.0140170)

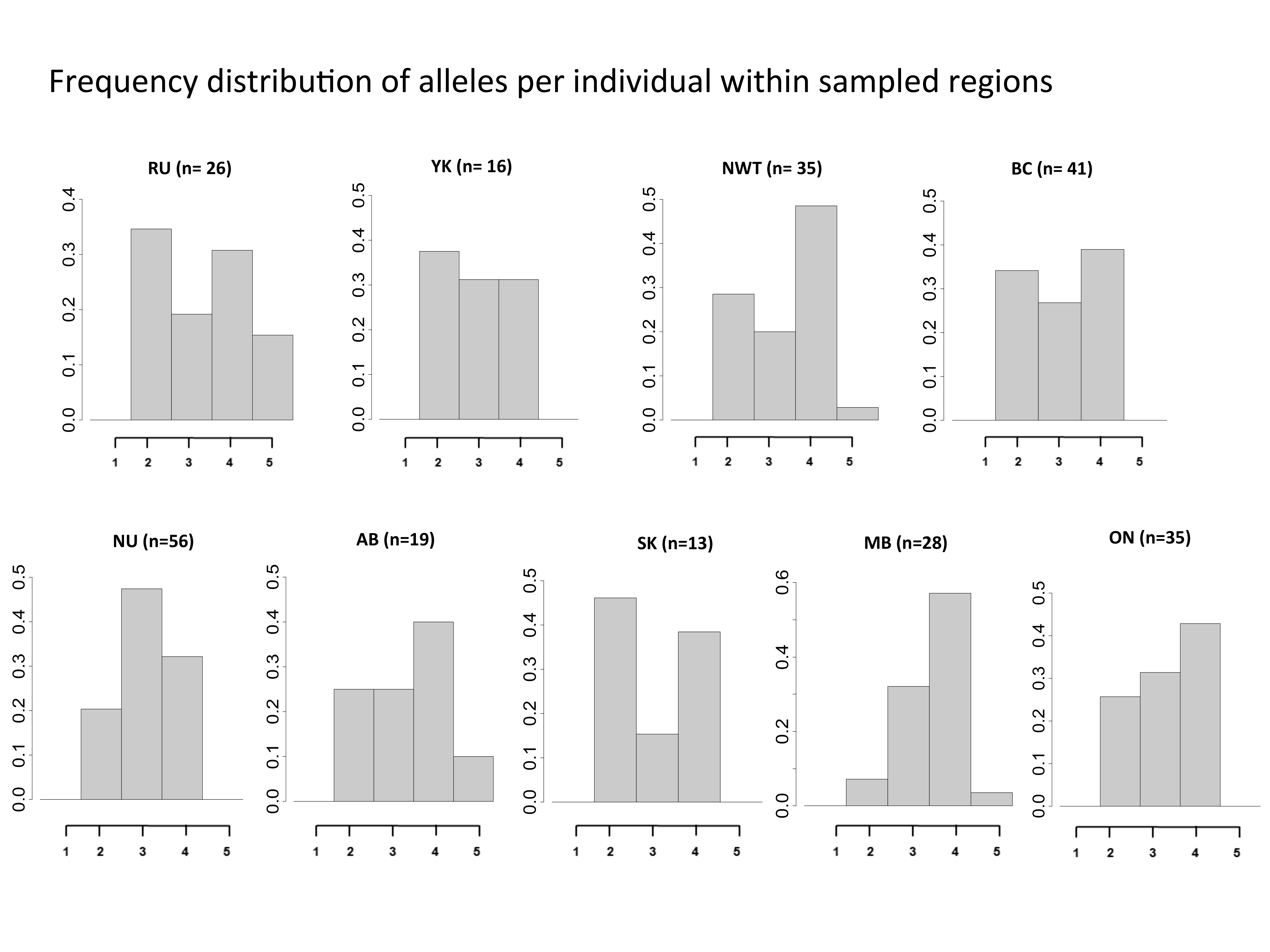

Supplement: S1 Fig — (TIF) [file pone.0140170.s001.tif]

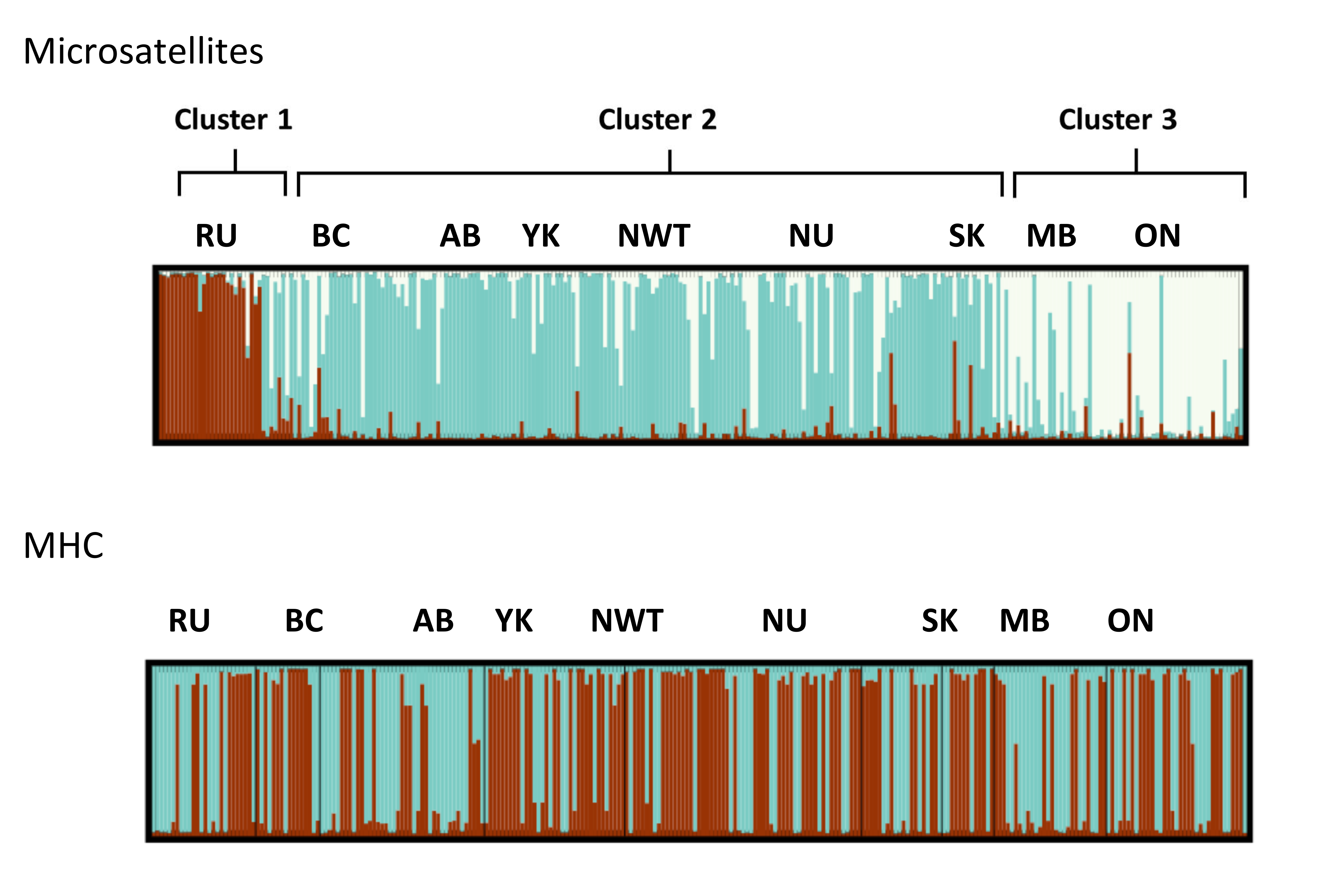

Supplement: S2 Fig — Although STRUCTURE identified k = 2 in the MHC data, there was no evident pattern of population genetic structure shown in the structure bar plot. (TIF) [file pone.0140170.s002.tif]
