# Supplementary material for: Lack of Spatial Immunogenetic Structure among Wolverine (Gulo gulo) Populations Suggestive of Broad Scale Balancing Selection
Source: PLoS One. 2015 Oct 8;10(10):e0140170. doi: 10.1371/journal.pone.0140170 (PMC4598017; doi:10.1371/journal.pone.0140170)
Supplement: S1 Table — (DOC) [file pone.0140170.s003.doc]

**S1 Table.** Population pairwise D*_EST_* values for eleven microsatellite loci (above the diagonal) and for MHC DRB-2 (below the diagonal) in nine sampling regions for wolverines. Regions abbreviations refer to text.

|  | **RU** | **YK** | **NWT** | **NU** | **BC** | **SK** | **AB** | **MB** | **ON** |
| --- | --- | --- | --- | --- | --- | --- | --- | --- | --- |
| **RU** |  | 0.127 | 0.203 | 0.258 | 0.269 | 0.16 | 0.167 | 0.241 | 0.32 |
| **YK** | 0.152 |  | 0 | 0 | 0 | 0 | 0 | 0 | 0.061 |
| **NWT** | 0.193 | 0 |  | 0 | 0.004 | 0 | 0 | 0.036 | 0.14 |
| **NU** | 0.159 | 0 | 0 |  | 0.049 | 0 | 0 | 0.073 | 0.168 |
| **BC** | 0.204 | 0.083 | 0.072 | 0.079 |  | 0.017 | 0 | 0.067 | 0.18 |
| **SK** | 0.211 | 0.033 | 0 | 0 | 0.137 |  | 0 | 0 | 0.109 |
| **AB** | 0.133 | 0 | 0.056 | 0.026 | 0.029 | 0.071 |  | 0.01 | 0.103 |
| **MB** | 0.21 | 0.081 | 0.054 | 0.055 | 0.01 | 0.053 | 0 |  | 0 |
| **ON** | 0.218 | 0.045 | 0.012 | 0.034 | 0.058 | 0.012 | 0 | 0 |  |
